# Assessing the feasibility of screening and providing brief advice for alcohol misuse in general dental practice: a clustered randomised control trial protocol for the DART study

**DOI:** 10.1136/bmjopen-2015-008586

**Published:** 2015-10-06

**Authors:** Antiopi Ntouva, Jessie Porter, Mike J Crawford, Annie Britton, Christine Gratus, Tim Newton, Georgios Tsakos, Anja Heilmann, Hynek Pikhart, Richard G Watt

**Affiliations:** 1 UCL Research Department of Epidemiology and Public Health, University College London, London, UK; 2 Faculty of Medicine, Department of Medicine, Imperial College London, London, UK; 3 Division of Health and Social Care Research, King's College London, Dental Institute, London, UK

**Keywords:** PUBLIC HEALTH, PRIMARY CARE, ORAL MEDICINE

## Abstract

**Introduction:**

Alcohol misuse is a significant public health problem with major health, social and economic consequences. Systematic reviews have reported that brief advice interventions delivered in various health service settings can reduce harmful drinking. Although the links between alcohol and oral health are well established and dentists come into contact with large numbers of otherwise healthy patients regularly, no studies have been conducted in the UK to test the feasibility of delivering brief advice about alcohol in general dental settings.

**Methods and analysis:**

The Dental Alcohol Reduction Trial (DART) aims to assess the feasibility and acceptability of screening for alcohol misuse and delivering brief advice in patients attending National Health Service (NHS) general dental practices in North London. DART is a cluster randomised control feasibility trial and uses a mixed methods approach throughout the development, design, delivery and evaluation of the intervention. It will be conducted in 12 NHS general dental practices across North London and will include dental patients who drink above the recommended guidance, as measured by the Alcohol Use Disorders Identification Test (AUDIT-C) screening tool. The intervention involves 5 min of tailored brief advice delivered by dental practitioners during the patient's appointment. Feasibility and acceptability measures as well as suitability of proposed primary outcomes of alcohol consumption will be assessed. Initial economic evaluation will be undertaken. Recruitment and retention rates as well as acceptability of the study procedures from screening to follow-up will be measured.

**Ethics and dissemination:**

Ethical approval was obtained from the Camden and Islington Research Ethics Committee. Study outputs will be disseminated via scientific publications, newsletters, reports and conference presentations to a range of professional and patient groups and stakeholders. Based on the results of the trial, recommendations will be made on the conduct of a definitive randomised controlled trial.

**Trial registration number:**

ISRCTN81193263.

**Strengths and limitations of this study**

- The brief alcohol advice intervention has been modified and applied for the first time to the setting of a National Health Service (NHS) general dental practice.
- The training programme was designed after extensive consultation with key stakeholders and was tailored to address the needs and circumstances of NHS dental professionals and their patients.
- This feasibility study will inform the design of a future definitive evaluative study.

## Introduction

Alcohol misuse is a significant public health problem with major health, social and economic consequences. Findings from the latest Health Survey for England show that approximately 24% of men and 18% of women exceed the current Department of Health alcohol consumption guidelines.^1^ The annual total cost of alcohol misuse to the UK economy is thought to be around £21 billion, including £3.5 billion per year in direct costs to the National Health Service (NHS).^2^

The identification of people who are drinking above the recommended guidelines and the provision of brief advice by NHS primary care professionals are important components of an alcohol control strategy. National Institute of Health and Care Excellence (NICE) guidelines recommend that primary care professionals should screen all patients for alcohol misuse.^3^ A variety of screening questionnaires have been developed for use in primary care settings including the Alcohol Use Disorders Identification Test (AUDIT); a shorter version of AUDIT containing the first three questions (AUDIT-C); Fast Alcohol Screening Test (FAST) and the Modified-Single Alcohol Screening Question (M-SASQ). A systematic review has shown that formal screening tools are reliable and valid for detecting alcohol misuse among patients attending primary care services.^4^ Brief interventions can take as little as 5–10 min to deliver and evidence suggests a single session is as effective as multiple sessions.^5^ Numerous systematic reviews and meta-analyses have reported positive outcomes of brief interventions delivered in various health service settings to reduce harmful drinking. The most recent Cochrane review of brief interventions reported significant reductions in weekly drinking at 1 year follow-up.^5^

The links between alcohol and oral health are well established. Most importantly these include increased risk for oral cancers, with and without the synergistic effect of smoking,^6^ and for dental trauma (fractured teeth) and facial injury either through accidental falls, road traffic accidents or interpersonal violence.^7^ In addition, excessive alcohol use is also associated with loss of tooth surface through dental erosion,^8^ halitosis and tooth staining.^9^ ^10^ In light of this, various policy guidelines have advocated the need for dental professionals to screen and provide brief advice to their patients.^11^ ^12^ Unlike many other parts of the NHS, a significant proportion of the general population routinely visits a dentist for check-ups and dental treatment. Data from the 2009 national Adult Dental Health Survey showed that 77% of adults in England reported having seen a dentist in the past 2 years.^13^ Dental professionals are therefore well-placed to provide brief alcohol interventions to the general adult population, many of whom may not have come into routine contact with other health professionals. Despite the serious implications of excessive alcohol intake on oral health and the potential of addressing the issue during a dental visit, very few studies have assessed the value of brief alcohol advice delivered by oral health professionals to their patients.

A limited body of research has been conducted on the use of certain screening tools to assess the prevalence of high-risk drinking among dental patients. Two clinical audits assessing the completion of an alcohol consumption question (How many units of alcohol do you consume each week?) in a standard medical history form used in the emergency clinic at the university dental hospital in Cardiff, found that the question was either completed incorrectly, or not used at all. In the second audit, the M-SASQ question (How often do you have eight or more standard drinks if male, or six or more standard drinks if female, on one occasion?) was answered more often and resulted in more patients receiving alcohol advice compared to the standard question.^14^ A Scottish study using the AUDIT screening tool reported that 31% of dental patients were drinking at harmful levels,^15^ while a US study found that 25% of dental patients in an emergency walk-in dental clinic were drinking at hazardous levels based on scores from the AUDIT-C tool.^16^ To date, there are no studies in the UK that assess the prevalence of higher risk drinking using the AUDIT-C questionnaire in general dental practices.

A limited number of randomised controlled trials have investigated the effectiveness of brief advice interventions in clinical dental settings, using dental care professionals rather than dentists. A randomised controlled trial in Cardiff used a nurse-led brief intervention utilising motivational interviewing with young males who were treated at an oral and maxillofacial surgery outpatient clinic for alcohol related facial injury and demonstrated a significant reduction in alcohol consumption in the intervention group compared to the controls at 12 months follow-up.^17^ The study results were replicated in a larger sample of men and women at three oral and maxillofacial surgery outpatient clinics in the West of Scotland with a significant reduction in days of drinking and heavy drinking in the intervention group compared to the control group at 12 months follow-up. The patients with the highest alcohol consumption benefited the most from the intervention.17a The team also demonstrated that the nurse-led brief motivational intervention was feasible in a maxillofacial trauma clinic.^18^ In the USA, a small scale motivational interviewing intervention with additional personalised feedback delivered by hygienists in dental practices demonstrated a significant reduction in total drinks per week in the intervention practices compared to controls at 6 months follow-up.^19^ However, there have not been any randomised controlled trials of brief alcohol advice in the UK undertaken in primary dental care, the setting where the vast majority of dental patients are seen.

Exploratory work has, however, revealed that general dental practitioners are reluctant to engage with patients about alcohol due to lack of confidence in discussing the subject and concerns that this may adversely affect the practitioner–patient relationship.^20^ More research is needed to determine the potential effectiveness of providing brief advice on alcohol misuse in general dental practice. In line with Medical Research Council (MRC) guidance on the development and evaluation of complex interventions, before conducting a definitive trial it is necessary to undertake a feasibility study^21^ in order to access the acceptability of the study components and intervention to patients and dental professionals.

## Aims and objectives

The Dental Alcohol Reduction Trial (DART) study aimed to assess the feasibility and acceptability of screening for alcohol misuse and delivering brief advice to patients attending NHS general dental practices in North London.

The study objectives were:

- To explore the views of dental professionals and dental patients on the relevance and importance of alcohol misuse to oral health, as well as the acceptability of screening and providing brief alcohol advice in general dental settings.
- To develop and evaluate a brief alcohol advice intervention tailored for use in NHS general dental practices and to assess through a process evaluation the feasibility and acceptability of the intervention to patients and dental professionals.
- To assess the feasibility of the main trial methodology including the use of a screening tool, subject recruitment and retention, and data collection procedures including economic evaluation to inform the future design of a definitive randomised controlled trial.

## Methodology

The DART study comprises of two phases:

- The developmental phase which involved separate focus groups with dental professionals and dental patients. This phase informed the development of the training programme and intervention for the main phase of the trial.
- The feasibility trial which involved: (A) engagement and recruitment of dentists, (B) training dental teams on research governance (control and intervention arms) and on how to deliver alcohol brief advice (intervention arm), (C) participant recruitment, (D) data collection (baseline and 6 months follow-up data), (E) process evaluation including assessment of intervention fidelity, acceptability to participants and dental professionals and feasibility of cost-effectiveness evaluation.

## Trial registration

The study is registered as a primary clinical trial (ISRCTN number: 81193263).

## Study design, setting and population

**Figure 1:**
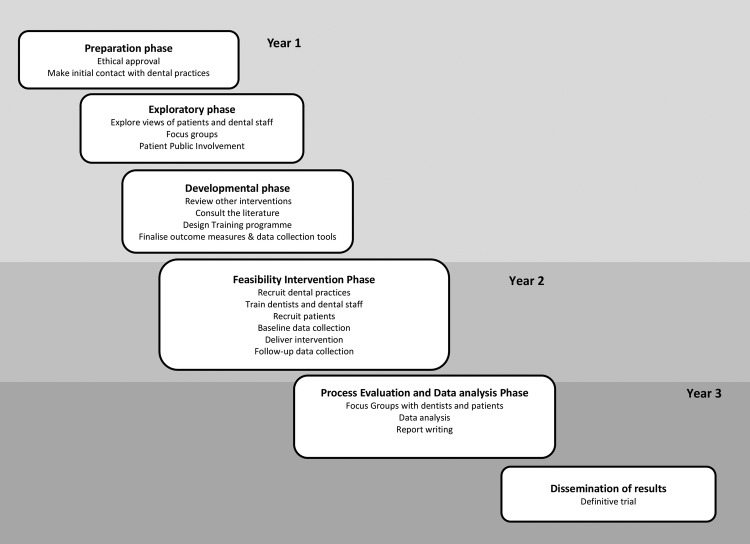
DART study timeline. DART, The Dental Alcohol Reduction Trial.

The DART study is a cluster randomised control feasibility trial and uses a mixed methods approach throughout the development, design, delivery and evaluation of the intervention. The trial took place in general dental practices across North London (Islington, Camden, Haringey, Brent, Barnet, Enfield and Redbridge). The study timeline is presented in figure 1.

## Development of the intervention

### Developmental phase

The developmental phase of the study involved the completion of five focus groups; two with dental professionals and three with dental patients. Recruitment for the developmental phase started in December 2013. The discussions explored dental teams’ understanding and views of the importance of alcohol misuse in relation to oral health; perceived extent of alcohol misuse among patients; dental professionals’ attitudes towards screening, providing advice, and referral when necessary; acceptability and barriers of engaging in alcohol screening and advice; best ways of delivery; and also perceived training and support needs.

Similarly, discussions with dental patients explored their views on the relevance of alcohol to oral health and dentistry, acceptability of dental teams enquiring about alcohol intake and offering advice, views on the best ways for dental teams to approach the subject and provide advice and support, and on suitable information for participants in the group not receiving the intervention. The information gathered during these discussions was used to design the intervention.

## Recruitment of dental practices

NHS dental practices were recruited into the study through a number of complementary ways. The principal investigator along with the research team had already established excellent links with a number of dental practices across North London, which had participated in previous research studies and had expressed interest in being involved in future research. In addition a mail was sent out to all NHS dental practices in the selected areas of North London and interested practices then visited by the principal investigator. Finally, a snowballing recruitment method was also used with dental professionals already engaged in the study recommending other colleagues who might be interested in participating in the research.

### Randomisation and blinding

To avoid risk of contamination, dental practices were randomised to intervention and controls. Lists of all the dental practices which expressed interest in participating in the trial were compiled and equal numbers allocated to each arm of the trial using simple randomisation. A member of staff not involved in data collection or delivering the intervention was responsible for allocating dental practices to groups. The researchers collecting the 6-month follow-up data will be masked to the practices’ allocation status.

### Dental teams’ training and support

All members of the dental teams who participated in the study (reception staff and dental professionals) at each practice were trained in research governance issues. This included how to introduce the study to potential participants, recruitment strategies, obtaining verbal and written consent, data collection procedures, confidentiality and data storing/handling.

All dentists from the intervention practices attended a 6 h training course over 2 days (table 1). The training sessions were run by the research team and an experienced alcohol trainer (RS), who has successfully delivered similar training to other healthcare professionals. The sessions were highly interactive with a combination of role plays and other exercises.

**Table 1:** Alcohol brief advice: training session plan

Training components	Description
Pretraining questionnaire	Assessment of the practitioners’ knowledge skills and attitudes towards providing alcohol advice in dental settings
Background epidemiology of alcohol	Building the practitioners’ theoretical knowledge on alcohol epidemiology, nationally and locally
Link of alcohol with health and social problems	Reviewing the impact of alcohol on the individual and the wider environment including financial burden to society and health services
Links between alcohol and oral health	Reviewing theoretical knowledge on the links between alcohol and oral health
Defining core terminology	Introduction to units of alcohol, current guidelines on drinking for men and women as well as defining levels of risk
Exploring the role of dental teams in providing alcohol brief advice	Identifying practitioners’ initial concerns regarding providing alcohol brief advice and explaining how the training programme will address these
Raising the issue of alcohol	Introducing the issue of alcohol in an appropriate manner in dental settings
The use of AUDIT-C screening tool	Theoretical background to the AUDIT-C tool and scoring system
Delivering brief advice	Video of GP delivering brief advice using the modified tool. Analysis of each part of the modified tool and the general leaflet
Tailoring advice for dental patients	Introducing specific skills in making the alcohol screening and brief advice relevant to the dental patient
Practising delivering brief advice	Role play which includes introducing the issue of alcohol, going through the AUDIT-C questionnaire and delivering brief advice using the tool to a series of compliant and resistant patients
Signposting services	Explaining the referral process and the way the issue of referral can be arisen with dental patients
Post-training questionnaire	Assessment of the practitioners’ knowledge skills and attitudes towards providing alcohol advice in dental settings
Next steps	Introducing the next parts of the DART study and arranging research governance training at each individual practice
Close	Summary and Q&A session

AUDIT-C, the Alcohol Use Disorders Identification Test; DART, The Dental Alcohol Reduction Trial; GP, general practitioner; Q&A, question and answer.

The first session provided the participants with essential theoretical knowledge on issues around alcohol, including its epidemiology, public health burden and links to oral health. Key terminology was defined (units of alcohol, current guidelines of alcohol consumption and levels of risk), followed by an introduction to raising the issue of alcohol and using the AUDIT-C screening tool. Exercises included estimating alcohol units in various popular alcoholic drinks and determining patients’ AUDIT-C score using real-life scenarios. During the second session, dental professionals were introduced to a specifically modified version of the brief advice tool tailored to oral health. Using role-plays of increasing complexity, dentists learnt how to raise the issue of alcohol with their patients, go through the AUDIT-C tool, provide feedback based on the AUDIT-C score and then proceed with delivering brief advice. Concerns and barriers to delivering the intervention were explored and addressed. Area specific information on local alcohol services for referral was also provided to each practice. The training programme was evaluated using a pretraining and post-training questionnaire which assessed participants’ knowledge, skills and attitudes towards screening for alcohol misuse and providing preventive advice.

## Patient recruitment, consent and data collection procedures

Different patient recruitment approaches were used by the dental professionals in the participating practices. Some practices used a targeted approach based on their knowledge of their patients, and others approached all patients attending for treatment during the recruitment period. Recruitment for the main trial phase began in May 2014. Dental staff at each participating dental practice were trained and briefed on effective ways of approaching potential participants.

### Inclusion criteria

Patients were eligible to take part in the study if they consumed alcohol above the current recommendations^22^ as assessed by the Audit-C screening tool, were aged 18 years or above, attended the dental practice and were able to speak, read and write in English sufficiently to complete the study questionnaires/interviews.

### Exclusion criteria

In addition, participants were excluded if they were already involved in a research study conducted in the dental practice and if they were already seeking or receiving help for alcohol dependence.

### Screening

From our exploratory work, it was ascertained that compared to the M-SASQ and FAST alcohol screening questionnaires, the AUDIT-C questionnaire^23^ was considered preferable by dental professionals and patients in terms of the time needed to complete the instrument and the nature of the questions included. The AUDIT-C is an effective and practical screening tool for detecting alcohol misuse in primary care settings.^24^ ^25^ To mask the focus on alcohol particularly for the participants in the control group, the screening questionnaire also included some more general lifestyle questions on diet and smoking, in addition to the AUDIT-C alcohol questions.

Patients who expressed an interest in participating in the study completed the screening questionnaire, which was then given to the dentist in order to ascertain eligibility for the study. A score of 5 or above in the AUDIT-C questions indicates drinking levels above the current recommendations and therefore eligibility to enter the study.^12^ After eligibility was ascertained, the dentist introduced the study. In the control arm the study was introduced as a lifestyle survey with no specific mention of alcohol. In the intervention arm, the study was introduced as a lifestyle survey but with a focus on alcohol in particular.

### Consent

Consent to participate was obtained in a two-stage process. Dental staff obtained verbal consent to screen participants using the AUDIT–C measure. Patients who obtained a positive AUDIT-C score were invited into the trial. Information sheets explained the details of the study and any queries were addressed at this point. If the participants were happy to continue, written consent was obtained at this point by the dental professional.

## Baseline measures

All eligible and consenting participants were asked to complete a short baseline questionnaire which comprised of basic demographic questions (age, sex and education level) and the EuroQoL five dimensions questionnaire (EQ-5D).^26^ The EQ-5D-5 L is a quality of life measure used extensively in economic evaluations. The tool divides health status into five dimensions (mobility, self-care, usual activities, pain/discomfort, and anxiety/depression). Each of these dimensions has five possible levels giving 3125 possible health states.

## Follow-up measures

Six months after the completion of baseline measures, all participants will be contacted via telephone by a researcher masked to the participant's allocation status. The full AUDIT tool will be administered.^24^ Alcohol consumption in the last 90 days will also be collected using the Form 90.^27^ This validated alcohol consumption tool provides a detailed day-by-day account of alcohol use in the 90 days prior to the interview. As in baseline, the health-related quality of life EQ-5D measure will also be completed^26^ along with a short patient satisfaction questionnaire on services received at the dental practice.

## Brief alcohol advice

Eligible participants attending the intervention practices were given up to 5 min of simple, structured, brief advice using a modified version of the brief advice tool ‘Brief advice about alcohol risk’ which was developed for the Screening and Intervention Programme for Sensible drinking (SIPS) study^28^ and is based on the Simple Structured Advice intervention tool and part of the UK version of the WHO collaborative Drink-Less brief intervention programme.^29^ This form of brief advice was shown to be as effective as more intensive and time consuming alcohol counselling.^5^ The intervention was tailored for use in a dental setting by highlighting the potential harm alcohol may cause to oral health. To support and reinforce the advice, the Change for Life leaflet ‘Don’t let fdrink sneak up on you’ was also given. The leaflet contained user friendly and concise information on the health risks of alcohol misuse, guidance on units and information on support services. Participants who scored above 10 on the AUDIT-C questionnaire were additionally given information on local alcohol services for advice and help.

**Figure 2:**
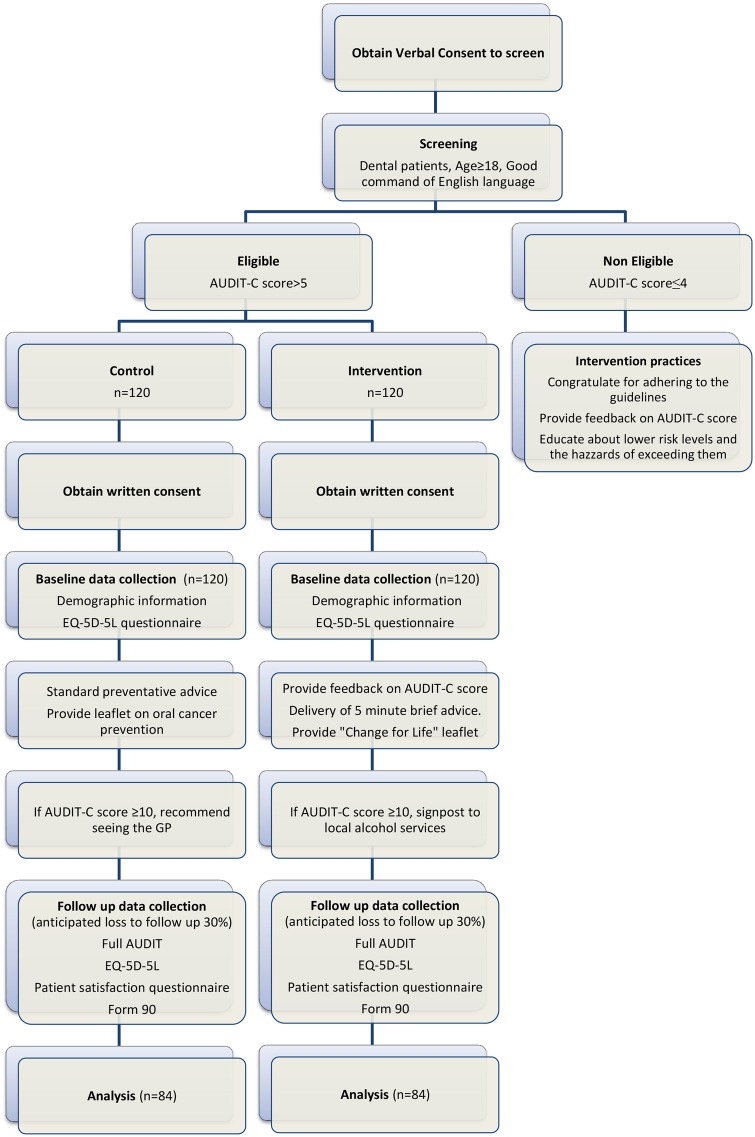
DART study flowchart. AUDIT-C, the Alcohol Use Disorders Identification Test; DART, The Dental Alcohol Reduction Trial.

In the control practices, eligible participants received standard oral healthcare and were initially given an oral cancer prevention leaflet which included brief guidance on reducing alcohol intake and stopping smoking. After all the follow-up data are collected, they will then also be offered the 5 min brief advice and given a copy of the Change for Life leaflet. A summary flow chart of the study process in control and intervention practices can be found in figure 2.

## Outcomes

We will measure recruitment and retention rates as well as practicality of engagement with dental practices, fidelity of delivery of the intervention and general acceptability of the study procedures from screening to follow-up. In addition, we will also assess if outcome measures proposed for the main trial could successfully be collected. The proposed primary outcome of the full trial is the score on the full AUDIT questionnaire, with a cut-off of 8 or more as used in the SIPS programme.^23^ Proposed secondary outcomes include mean weekly units of alcohol consumed during the previous 90 days using the Form 90^27^ average drinks (8 mg of pure ethanol) per day, health-related quality of life measure using EQ-5D^26^ for the economic evaluation calculations, and questions relating to satisfaction with dental services taken from the Adult Dental Health Survey 2009.^13^ Rates of data completeness for the proposed outcomes will also be evaluated.

## Sample size

For feasibility studies a detailed sample size calculation is neither appropriate nor necessary. The primary aim of conducting feasibility trials is to provide data to inform power calculations for a future larger scale trial.^30^

Based on pragmatic considerations it was estimated that 12 dental practices were required with a sample of 240 participants at baseline—20 participants per practice. Assuming a 30% drop out rate this would give a sample of 168 participants at the 6 months follow-up. This figure was deemed to be reasonable as the average dental practice has approximately 1500 adult patients of whom approximately 25% were drinking at higher risk levels^15^ and if 60% of the patients screened agreed to participate.

No published studies have reported the effect of brief advice provided in general dental practice. The variance in the proposed primary outcome for the definitive trial (AUDIT score at 6 months) will be used in conjunction with recruitment and follow-up rates as well as an estimate of the intraclass correlation coefficient (and 95% CIs) in order to calculate the sample size for the definitive trial.

## Patient and public involvement

To ensure that the study was relevant and informed by patient experience, a dental patient research forum was established in one of the dental practices involved in the study. The principal dentist invited a diverse mix of dental patients to join the forum. Semistructured discussions took place with the forum members. These discussions were facilitated by the patient and public involvement co-investigator (CG) and members of the study team. The forum was consulted on a variety of practical aspects of the study, such as the design of recruitment materials and data collection questionnaires, the best ways to introduce the study to potential participants, suggestions on retention of the study sample and effective follow-up methods. The group also assisted with piloting the data collection questionnaires once they were finalised.

## Data monitoring procedures

The research team developed a comprehensive monitoring system with monthly visits to the dental practices to collect the screening and baseline questionnaires and to ascertain if recruitment and data collection were going well. Visit checklists were designed and completed at each visit and feedback emails were sent when deemed useful for the practitioners.

## Process evaluation

The process evaluation will consist of a mixed methodology using both qualitative and quantitative methods. This will include an assessment of the recruitment procedures, satisfaction among participating dental patients and dental teams, and intervention fidelity.

### Intervention fidelity

All dentists at the intervention practices were asked to complete a fidelity form for each patient who received the brief intervention to ensure that the intervention was delivered consistently. The form was based on a checklist used in a previous trial,^31^ covered all components of the brief advice delivered, and included a question asking how long it took to deliver the intervention.

### Interviews with patients and dental professionals

Face to face individual interviews will be held in the intervention and control dental practices to gain an understanding of the dental professionals’ experience and views of the study. Dentists and reception staff who were directly involved with the recruitment and data collection process will be interviewed by the research team on an individual basis. The discussions with the intervention practices will assess views on the recruitment procedures, value and relevance of the alcohol brief advice training, the appropriateness of delivering advice on alcohol in the dental practice and overall perceptions surrounding participation in research. In the control practices, the discussions will focus on the study protocol procedures namely the challenges and facilitators in recruiting dental patients for the study and the data collection process. In addition, dentists in the control arm will be asked if they would find the training for delivering alcohol advice relevant and useful to them.

The experience of participants will also be evaluated through individual telephone interviews and will focus on their experiences of the study (the recruitment and data collection process, their thoughts on the delivery of the alcohol brief advice by the dentist, whether the advice had an impact on their alcohol consumption) and general perceptions around participating in research. Members of the research team will also be interviewed one-to-one by an external interviewer, discussions surrounding recruitment of dental practices, training of dental teams and general perceptions of the organisation of the study will be carried out.

## Planned data analysis

We will assess key feasibility parameters, such as recruitment and retention rates, number of participants screened in order to assess eligibility, the practicalities of screening and delivering the intervention in dental settings, in order to inform the acceptability of the study procedures in both patients and dental professionals. The study instruments will be evaluated in terms of ease of administration as well as acceptability by participants in order to inform their suitability for the main trial. Descriptive analysis of the proposed main outcome measure (AUDIT score) will provide a SD which will be subsequently used in the sample size calculation for the definitive trial.

The process evaluation interviews with dentists, dental teams, dental patients and members of the research team involved in the study will be transcribed and coded, classified and organised into main themes using thematic analysis.

A decision to process to the full scale study will be based on recruitment of at least 85% of the planned sample within the allocated time, completion of primary outcome follow-up interviews with no less than 75% of participants and receipt of brief advice by at least 60% of those randomised to the intervention arm.

## Discussion

Alcohol misuse remains a major health and social problem in the UK affecting a significant proportion of the population. Excessive alcohol consumption adversely affects oral health in a variety of ways, but currently most dentists do not ask their patients about their alcohol intake or provide any advice to those that require support. Indeed, many health professionals lack the necessary knowledge and confidence to discuss alcohol misuse with their patients.^32^ Previous studies have shown that dentists were also concerned about their lack of knowledge in this field and worried that they might alienate their patients by raising the topic of alcohol.^20^ Dentists working in primary care are, however, in an ideal position to offer brief advice to their patients, many of whom will not have contact with any other health professional. Despite this opportunity very few studies have developed and evaluated alcohol brief advice interventions in primary dental care settings.

This paper has described the study methodology and intervention design for an alcohol screening and brief advice intervention delivered in NHS dental practices in North London. The comprehensive feasibility study will provide detailed insights into the development, implementation and evaluation of the alcohol intervention which will inform the design of a future definitive trial. Findings from the initial exploratory phase of the study will help ensure that the intervention training programme and resources are tailored to the needs and circumstances of NHS dental professionals.

Furthermore, the feasibility trial will explore the logistics and practicality of delivering brief advice to dental patients in a NHS dental setting. The planned process evaluation will determine the acceptability of the intervention to dental professionals and patients. These implementation and feasibility issues will be explored in depth as they will likely influence uptake of this approach in primary dental care. The comprehensive methodology of this study will provide useful data on this important and under researched topic and provide evidence which will inform primary dental care practice in and outside of the UK.
